# A community-based cluster randomised trial of safe storage to reduce pesticide self-poisoning in rural Sri Lanka: study protocol

**DOI:** 10.1186/1471-2458-11-879

**Published:** 2011-11-21

**Authors:** Melissa Pearson, Flemming Konradsen, David Gunnell, Andrew H Dawson, Ravi Pieris, Manjula Weerasinghe, Duleeka W Knipe, Shaluka Jayamanne, Chris Metcalfe, Keith Hawton, A Rajitha Wickramasinghe, W Atapattu, Palitha Bandara, Dhammika de Silva, Asanga Ranasinghe, Fahim Mohamed, Nicholas A Buckley, Indika Gawarammana, Michael Eddleston

**Affiliations:** 1 Clinical Pharmacology Unit, University of Edinburgh, The Queen's Medical Research Institute, 47 Little France Crescent, Edinburgh, EH16 4TJ, UK; 2 Department of International Health, Immunology and Microbiology, Faculty of Health Sciences, University of Copenhagen, Copenhagen, Denmark; 3 School of Social and Community Medicine, University of Bristol, Canynge Hall, 39 Whatley Road, Bristol, UK; 4 South Asian Clinical Toxicology Research Collaboration (SACTRC), Faculty of Medicine, University of Peradeniya, Peradeniya, Sri Lanka; 5 Drug Health, Royal Prince Alfred Hospital, Sydney, Australia; 6 Faculty of Medicine, University of Kelanyia, Kelanyia, Sri Lanka; 7 Centre for Suicide Research, Department of Psychiatry, University of Oxford, Oxford, UK; 8 Provincial Department of Health Services, North Central Province, Anuradhapura, Sri Lanka; 9 Department of Pharmacy, Faculty of Allied Health Sciences, University of Peradeniya, Peradeniya, Sri Lanka; 10 Professorial Medicine Unit, POW Clinical School, University of New South Wales, Sydney, Australia; 11 Department of Medicine, Faculty of Medicine, University of Peradeniya, Peradeniya, Sri Lanka

## Abstract

**Background:**

The WHO recognises pesticide poisoning to be the single most important means of suicide globally. Pesticide self-poisoning is a major public health and clinical problem in rural Asia, where it has led to case fatality ratios 20-30 times higher than self-poisoning in the developed world. One approach to reducing access to pesticides is for households to store pesticides in lockable "safe-storage" containers. However, before this approach can be promoted, evidence is required on its effectiveness and safety.

**Methods/Design:**

A community-based cluster randomised controlled trial has been set up in 44,000 households in the North Central Province, Sri Lanka. A census is being performed, collecting baseline demographic data, socio-economic status, pesticide usage, self-harm and alcohol. Participating villages are then randomised and eligible households in the intervention arm given a lockable safe storage container for agrochemicals.

The primary outcome will be incidence of pesticide self-poisoning over three years amongst individuals aged 14 years and over. 217,944 person years of follow-up are required in each arm to detect a 33% reduction in pesticide self-poisoning with 80% power at the 5% significance level. Secondary outcomes will include the incidence of all pesticide poisoning and total self-harm.

**Discussion:**

This paper describes a large effectiveness study of a community intervention to reduce the burden of intentional poisoning in rural Sri Lanka. The study builds on a strong partnership between provincial health services, local and international researchers, and local communities. We discuss issues in relation to randomisation and contamination, engaging control villages, the intervention, and strategies to improve adherence.

**Trial Registritation:**

The trial is registered on ClinicalTrials.gov ref: NCT1146496 (http://clinicaltrialsfeeds.org/clinical-trials/show/NCT01146496).

## Background

Pesticide poisoning is a major public health in rural Asia [1] and a significant burden on health services [2]. In a recent systematic review the global estimate of deaths due to pesticide self-poisoning was between 250-370,000 each year [3]. The WHO recognises pesticide poisoning to be the single most important global means of suicide and has established an initiative aiming to reduce the number of deaths [4]. Several approaches have been proposed to reduce mortality from pesticide self-poisoning [5-7].

Restricting easy access to pesticides in rural households to prevent their use in impulsive self-harm has become a popular recommendation [8-13]. There is convincing evidence that restricting access to commonly used, highly lethal methods of suicide not only reduces method-specific suicide rates, but can also significantly reduce overall rates [8,13-15]. Some of the factors that have been highlighted as determining the impact of modifying access to means include the popularity of the method, the danger associated with the method, levels of impulsivity, the risk of method substitution, and the ease with which it can be implemented [15].

Pesticide self-poisoning is prevalent in Sri Lanka; it is the most common method of suicide [16], is highly lethal [17], and is associated with impulsivity [5,18,19]. One possible consequence of restricting access to pesticides is the substitution of means of self-harm. This might offset the beneficial impact of means restriction if the newly adopted method is more accessible and of higher lethality. However, such effects have not previously been seen in Sri Lanka: the fall in deaths from pesticide self-poisoning seen since 1995 has not been associated with large increases in other methods of suicide [16].

The pesticide industry has long argued for secure storage and the use of locked boxes to prevent pesticide poisoning, and has started projects testing and scaling up the use of safe storage boxes [20]. It has supported a number of meetings on this option in Singapore, Durban, and Geneva [21,22].

**Figure 1:**
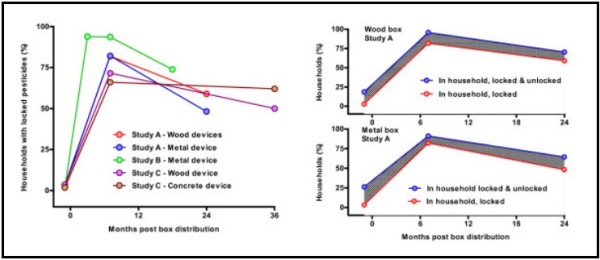
**Household storage of pesticides in pilot studies in Sri Lanka**. **Left - **% of households storing pesticides safely in or around their house in the five studies. **Right - **Comparison of the % of households in Study A storing pesticides in their household (top line) and the % storing pesticides safely locked up (bottom line) after provision of a safe storage container. The hatched area represents households storing unlocked pesticides. ***Note***, there was no change in the number of households that stored pesticides in the house without locking them away. Instead, the intervention markedly increased the absolute number of households storing pesticides in the house, potentially increasing the risk of poisoning if the compliance with locking them away fell away over time.

There have been several small scale pilot projects with high levels of community acceptability (Table 1) [23-25]. Whilst this approach appears to make good sense, the provision of containers may actually increase the storage of pesticides in or close to the household rather than at a distance in the field thereby increasing the risk of pesticide ingestion at times of stress. Pilot studies in Sri Lanka showed that this indeed happened, with household storage of pesticides (whether locked or unlocked) increased from 0% to between 75-98% over the first few months after provision of the boxes (Figure 1).

**Table 1:** SACTRC and Oxford Centre for Suicide Research (OSCR) safe storage pilot studies in Sri Lanka

Study	Start	Containers	Households*	Follow-up
A. SACTRC [23]	May 2005	• Wood in-house container • Metal in-house container	116 56	24 months
				
B. OCSR [24]	March 2006	• Metal in-house container	362	18 months
				
C. SACTRC [25]	May 2006	• Wood in-house container • Concrete in-field container	53 103	7 months
				

*Households that received particular containers and were using pesticides at 7 months

Although in one of the studies 82% of containers were locked securely at 7 months, by 24 months this had dropped to 55% [23].In addition, the use of the lock may decline over time and households may find it difficult to keep the key hidden from younger household members. Two further studies showed similar patterns in the drop- off in compliance after 12 months [24,25]. So, before this approach is implemented more widely a large scale community trial is required to determine its effectiveness and safety [26].

## Methods/Design

### Study hypothesis

The main study hypothesis is that the introduction of safe storage boxes to households who use or store pesticides will reduce the incidence of pesticide self-poisoning.

### Design

This is a community-based; cluster randomised controlled trial (RCT) of safe storage containers.

### Setting

**Figure 2:**
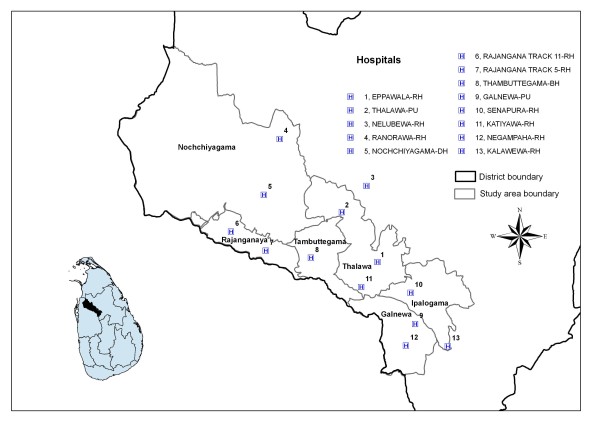
**Distribution of study areas and hospitals in the Anuradhapura District of Sri Lanka**. Five study areas divisional secretariats (Thambuthegama, Talawa, Galnewa, Rajanganaya and Nochchiyagama) and additional neighbouring division of Ipalogama where additional households may be recruited. The map also shows the fifteen peripheral hospitals (Rural, District, Primary and Base hospitals) within, or neighbouring, the study area that are used to identify cases of poisoning and self-harm.

We have set up the community-based cluster randomised control trial in the Anuradhapura district of Sri Lanka's North Central Province (population: 1,104,664: Census 2001). We plan to recruit approximately 162 villages primarily from the Mahaweli H region, including the divisional secretariats of Thambuthegama, Talawa, Galnewa, Rajanganaya and Nochchiyagama, (total population about 200,000) in the South East area of the district (Figure 2). The trial started recruiting households on 31 December 2010.

This irrigated rural region uses large amounts of pesticides, has high rates of self-poisoning [1,17,18] and was the location of earlier pilot safe storage trials [23-25]. The villages recruited to the pilot studies will be excluded from the current trial.

### Inclusion and exclusion criteria

All villages within the five Divisional Secretariats will be eligible for study entry except those recruited to our previous pilot studies. If necessary, additional households will be recruited from Ipalogama, a neighbouring divisional secretariat, to ensure an adequate number of households are enrolled. Those households in the intervention arm where farm workers are resident and where pesticide use or storage is reported will be eligible for a lockable safe storage box. Households where there is no adult available to provide consent will be excluded.

### Recruitment and baseline survey

An estimated one hundred and sixty-two villages are being recruited from five divisions of Anuradhapura district. The recruitment of villages is being undertaken in a rolling programme across the study area and the five Divisional Secretariats' will be divided roughly in half, creating 10 bands. All households will be approached and given a brief introduction and the householders' invited to verbally consent to participation.

A census is being performed, collecting baseline demographic (name and age/date of birth of all adults and children), socio-economic status, pesticide usage, previous self-reported self-harm and alcohol use data from each participating household. The questionnaire was tested in surrounding villages to ensure the questions were easily understood and the responses captured the relevant options in the local context. The data is being collected using a Juno Trimble survey device (http://www.trimble.com/junosb.shtml), allowing on-going quality assurance, monitoring of survey coverage, and recording of GPS coordinates for later mapping using a geographical information system. If a household refuses to participate only their location is recorded to ensure they are excluded from further follow up.

By collecting baseline survey data into a handheld recorder, transcribing errors are avoided by then downloading data directly into the main study database. In order to assess the accuracy of the data obtained, key data items (age and sex of each household member, quality of housing, and ownership of vehicles.) are validated by data collection managers revisiting a randomly selected (2%) sample of households in each village. The questionnaire is administered in Sinhala by a team of data collectors (high school graduates) recruited from the surrounding areas and trained in field research techniques and use of the questionnaire. Supervisors are present in the field to address any issues when administering the questionnaire or other issues arising from the fieldwork. Subsequent retraining is undertaken on a monthly basis to ensure compliance with the study protocol. Data is managed independently of the field team in Colombo and will be checked using a number of queries in Microsoft Access to improve accuracy.

### Randomisation

In this cluster randomised trial, villages (or groups of adjoining villages where boundaries are blurred; see Discussion) are the randomisation unit and are allocated to either the intervention or comparison group. Large imbalances between study arms in the number of allocated villages, the number of villagers in households eligible for a box, and the previous history of pesticide self-harm in the village, are avoided by the method of minimisation. Prior to allocation the village is added to each arm in turn and the differences on the three minimisation variables calculated. The odds of randomly allocating that village to one arm rather than the other is then modified to give an improved chance of the allocation, which achieves better balance between the two arms.

Steps have been taken to avoid selection bias during random allocation: (i) Villages are only randomised once household demographic data has been collected from all villages in the study administrative band; after this has been done villages cannot be withdrawn from or added to the study; (ii) The UK-based study statistician, who does not come into contact with study villages or data collectors, uploads data on the minimisation variables to an automated computer randomisation programme. Hence allocation concealment is ensured, it neither being possible to predict allocations, nor to withdraw or recruit villages or households once the allocation is known.

### Trial intervention

**Figure 3:**
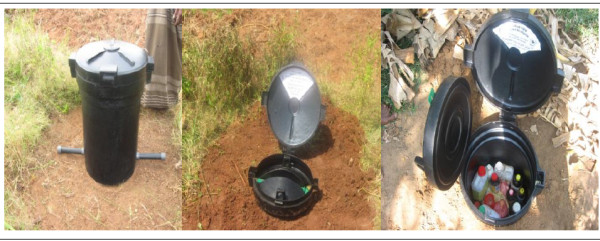
**Safe storage device for agrochemicals in Sri Lanka**. The UV-resistant plastic pesticide storage container to be used in this study. A) Device before installation: the metal bar at the base secures the device from theft. B) Device buried with two lids to protect the padlock from weather damage. C) Device can store several large and small bottles of pesticides. *Copyright Ravi Peiris*.

Following several pilot studies [23-25] of the acceptability and use of safe storage devices, we developed and field tested a storage container made from UV-resistant plastic that can be placed outside the house (in the home garden or field) and partially buried underground. Lock damage and corrosion were identified as a problem in the pilot studies. We have therefore designed the container to have two lids: an inner lid that can be locked and an outer black lid to protect the lock against weather or soil damage (Figure 3).

Following randomisation the GN official (lowest level of civil administration) in the villages in the intervention arm is informed and arrangements made for distribution of the devices to eligible households. A small demonstration is given and a range of materials are utilised to ensure the box is installed and used correctly. Efforts are made to ensure that the boxes are installed in field, compound or house and members of the distribution team follow up in the village to ensure all eligible people receive a box. Where the distribution team is uncertain about the eligibility of households they are required to bury the device to reduce the likelihood of contamination. Further promotion after distribution is limited to six monthly activities of limited scale in the villages.

As an evaluation of intervention adherence, a small sub-study in approximately five villages will be undertaken to examine the use of the safe storage device. Household opinions on the usefulness of the devices will be elicited through a survey and focus groups. In addition, the demographic survey will be repeated in each village at the end of the study to better estimate person years of follow-up and to observe whether the containers are still being used.

### Outcomes

Our primary and secondary outcome measures are objective. The primary outcome is the incidence of pesticide self-poisoning, both fatal and non-fatal, amongst villagers aged 14 years or older, over the three-year study period. Secondary outcomes will include the incidence of: pesticide poisoning in general (deliberate and accidental, all ages), self-harm (all methods, both fatal and non-fatal), self-poisoning (all substances) and pesticide poisoning in children (younger than 14 years).

Data on cases of fatal and non-fatal pesticide self-poisoning, accidental poisoning and self-harm will be prospectively collected from several sources:

• *Hospitals: *Patients admitted to hospital will be identified routinely at Anuradhapura Teaching Hospital (where SACTRC have an ongoing cohort study). Clinical research assistants have been employed to attend daily ward rounds in the four medical wards and weekly checks of admission books in Surgical, Pediatric, Intensive Care wards and the Morgue at Anuradhapura Teaching Hospital; they will be unaware of the village allocation. All rural peripheral hospitals in the district have been visited at least every month by researchers in a previous project (trial registration number ISRCTN73983810) to identify poisoning admissions; this will be continued in 13 hospitals in our study area. Field research officers will visit every peripheral hospital in the study area and neighboring hospitals to identify cases through checks of the admission and transfer books and discussion with relevant staff.

• *Community: *We will visit a sample of villages (10%) at least every two months to interview local key informants including the GN official, public health midwife and local health committee members (Suwasahana). These local committees will assist in linking cases to their households. If this approach improves the linking of cases to their households, we will seek to work with further community health committees during follow-up.

• *Coroners: *Deaths that occur before hospital presentation will be identified through review of the district coroners' and magistrates' records, as has been documented in another study [27].

Community-based studies in Sri Lanka indicate that practically all patients with poisoning are taken to hospital (J Maracek, unpublished data) and about 80% are transferred to the tertiary hospital [28]. Health care is free and usually available within one hour, and few barriers exist to hospital presentation [29].

The linking of individual cases of self-poisoning and self-harm back to the census data collected from households is complex due to imprecise demographic information. There are complex naming practices, poor recollection of birth dates and no unique identification numbers. Therefore a computer algorithm will match cases to census data using partial names and homophones, approximate ages and partial addresses to generate a list of possible matches that will then be checked manually.

### Pre-specified statistical analysis plan

The primary analysis will follow the intention-to-treat principle, comparing the observed incidence of self-harm using pesticides between individuals in villages allocated to the intervention, and individuals in villages allocated to the control arm. A Poisson regression model will be employed, with the standard errors inflated to accommodate the clustered design. This analysis will be adjusted for minimisation variables used in the random allocation and for seasonal variation in the incidence of pesticide self-poisoning. This same approach will also be used for the secondary outcome measures.

Pre-specified subgroup analyses will investigate whether the effectiveness of the safe storage intervention is modified by the village-level historical rate of self-poisoning (established in the baseline survey) and proportion of households provided with a locked box. We will perform a sensitivity analysis excluding the five intervention villages where sub-studies are undertaken as their participation may increase household compliance with safe storage.

### Sample size

Our previous research has found the incidence of self-poisoning in the district to be approximately 350 per 100,000 per year [3,5]. Fifty per cent of episodes involve pesticides - an incidence of 175 per 100,000 per year, or 525/100,000 over the three years of the study. We hypothesize that provision of pesticide storage containers might reduce the incidence of pesticide self-poisoning by 33%, from 175/100,000 to 117/100,000. At 80% power and 5% type I error rate, a total of 68,676 person years of follow-up are required in each arm of the study to detect a true intervention effect of this size.

The sample size for this study needs to be increased to allow for variation in self-harm rates between villages (i.e. the randomisation unit). Using our data on pesticide poisonings in 189 Sri Lankan villages [3], we estimate the design effect, or sample size inflation factor, to be at least 1.64. Compensating for clustering with a design effect of 1.75 (to allow for error in our estimate), 120,183 person years are required in each arm of the trial. To achieve this number of years of follow-up, for each trial arm 40,061 individuals must be followed for an average of three years.

However, some households within intervention villages will not use pesticides (and therefore not be offered a container), while others will not use their container (non-compliance). Some households within control villages may acquire a lockable container (contamination). If 20% of individuals in the intervention arm live in households not using a lockable container, and 5% of individuals in the control arm live in a household using a lockable container, then 217,944 person years of follow-up are required in each arm of the trial to compensate (24,216 households; 81 villages per arm; total 162 villages). Our assumptions about the number of households per village, the average number of household members, and the proportion of eligible households in each village, will be monitored as the baseline survey proceeds, and compensatory action (increasing the sample size) will be taken if deviations from those assumptions are likely to challenge the statistical power achieved.

### Study governance

Ethics approval was granted from the University of Peradeniya, Sri Lanka in March 2008, with amendments in January and July 2011. The Provincial Department of Health Services and national Ministry of Health have given their support to the study.

An independent Data Monitoring Committee (DMC) has been established for the trial. Interim analyses will be supplied by the trial statistician, in strict confidence, to the DMC, together with any other analyses the DMC may request. The DMC will recommend (i) continuation, (ii) modification, or (iii) cessation. Adverse events such as becoming aware of cases of poisoning using pesticides taken from a storage device within the home compound will be reported to the DMC immediately.

## Discussion

This study is a large community-based cluster randomised control trial of safe storage devices for agricultural pesticides. The results of the trial will establish the effectiveness of providing 'safe storage' containers for agrochemicals to rural Asian households to reduce intentional and accidental poisoning. They will be highly relevant for other parts of rural Asia and should allow policy makers to judge the usefulness of implementing this intervention.

The trial is only possible because a long-term collaboration between the Provincial Department of Health and SACTRC researchers has provided a platform on which to base this large community study. However, there are many issues with conducting the study, some of which are discussed below.

### Unit of randomisation and contamination

To reduce contamination (i.e. use of secure storage in the control arm) due to the exchange of boxes between households, especially those with family connections, this is a cluster randomised trial with village as the unit of randomisation. We had initially planned to use GN boundaries as a proxy for villages, but we found that in some areas there was considerable communication between households in neighbouring GN divisions, and household allegiances which reflected historical rather than current boundaries. In such cases we will combine several GN divisions into a single randomization unit to further reduce the scope for contamination. Where contamination does occur, it could result in the public health impact of the intervention being underestimated.

### Engaging control villages

At the end of the study, unless there is evidence that the containers are harmful, households in control villages will be offered a safe storage container. Focus groups were carried out before the study started to determine whether this offer is sufficient to encourage villages to enter the study despite the risk of being randomised to no intervention. These focus groups indicated that villagers consider that the offer of a free safe storage container, together with the great importance of pesticide poisoning to their communities, to be sufficient to encourage villages to enrol.

### Nature of the intervention

The original protocol included the choice of two devices, an in-ground device for the home-garden or field and a second device to be attached to a wall within the home. However, it was considered a safety risk to bring pesticides into the home where they had previously been stored outside, so it was decided not to distribute the second device. Participants are encouraged to install their device in the field or the furthest corner of their home garden. However, they can choose to install the device in their home.

### Adherence to intervention

The current study design is an effectiveness study which will assess the effect of the intervention (safe storage device) in a near 'real-life' situation to ensure that such an intervention can be extrapolated to rural communities elsewhere in Asia. In order to ensure that the device is properly used the field team provide village level demonstrations of installation and use of the device. The field team will also audit installation within 2 months to confirm the device has been installed and record its location. Help will be made available to those householders who find installation difficult, e.g. the elderly. However, to improve device use with minimal resources, we are planning to provide community level meetings every quarter in all villages; intervention and control, to encourage the local population to keep their pesticides stored safely.

## Competing interests

KH received financial support from Syngenta for a pilot study of safer storage of pesticides. ME has received financial support to attend a scientific meeting of a study funded by Syngenta. NAB and AHD have received travel expenses from Syngenta (a manufacturer of paraquat and some other pesticides) to attend meetings of a scientific advisory group in relation to studies of new paraquat formulations.

## Authors' contributions

ME, FK and AHD conceived the study based on FK's pilot work. ME, FK, DG, CM, AHD, KH, RP, and MW contributed to the original proposal from which this protocol was developed. MP, FK, ME, AHD, DG, CM, KH, SJ, RW, IG, WA, PB, DD, AR and FM are responsible for the conduct of the trial with ME as principal investigator and FK as chief scientist. NB is the chair of the DMC and has reviewed the protocol and the grant application from an early stage. MP is responsible for the day to day management of the trial and RP, MW and DK lead the fieldwork teams. MP and DK drafted the first version of the manuscript based on the grant application written by ME; MP collated contributions and subsequently redrafted the manuscript. All authors read and approved the final version.

## Funding

The work is supported by the Wellcome Trust (GR090958). ME is a Scottish Senior Clinical Research Fellow (Scottish Chief Scientist Office/Scottish Funding Council) and Lister Research Prize Fellow (Lister Institute for Preventative Medicine). DG and KH are both National Institute for Health Research Senior Investigators.

## Pre-publication history

The pre-publication history for this paper can be accessed here:

http://www.biomedcentral.com/1471-2458/11/879/prepub
